# Clinical Course and Gross Pathological Findings in Wild Boar Infected with a Highly Virulent Strain of African Swine Fever Virus Genotype II

**DOI:** 10.3390/pathogens9090688

**Published:** 2020-08-22

**Authors:** Antonio Rodríguez-Bertos, Estefanía Cadenas-Fernández, Agustín Rebollada-Merino, Néstor Porras-González, Francisco J. Mayoral-Alegre, Lucía Barreno, Aleksandra Kosowska, Irene Tomé-Sánchez, José A. Barasona, José M. Sánchez-Vizcaíno

**Affiliations:** 1 VISAVET Health Surveillance Centre, Complutense University of Madrid, 28040 Madrid, Spain; estefaca@ucm.es (E.C.-F.); agusrebo@ucm.es (A.R.-M.); nestorpo@ucm.es (N.P.-G.); fjmayoral@ucm.es (F.J.M.-A.); lbarreno@ucm.es (L.B.); alkosows@ucm.es (A.K.); aitome@ucm.es (I.T.-S.); jbarason@ucm.es (J.A.B.); jmvizcaino@ucm.es (J.M.S.-V.); 2 Department of Internal Medicine and Animal Surgery, Faculty of Veterinary Medicine, Complutense University of Madrid, 28040 Madrid, Spain; 3 Department of Animal Health, Faculty of Veterinary Medicine, Complutense University of Madrid, 28040 Madrid, Spain

**Keywords:** African swine fever virus, *Sus scrofa*, gross lesions, infection course, disease surveillance

## Abstract

African swine fever (ASF) is a notifiable disease that in recent years has spread remarkably in Europe and Asia. Eurasian wild boar (*Sus scrofa*) plays a key role in the maintenance and spread of the pathogen. Here we examined gross pathology of infection in wild boar with a highly virulent, hemadsorbing genotype II ASF virus (ASFV) strain. To this end, six wild boars were intramuscularly inoculated with the 10 HAD50 Arm07 ASFV strain, and 11 wild boars were allowed to come into direct contact with the inoculated animals. No animals survived the infection. Clinical course, gross pathological findings and viral genome quantification by PCR in tissues did not differ between intramuscularly inoculated or contact-infected animals. Postmortem analysis showed enlargement of liver and spleen; serosanguinous effusion in body cavities; and multiple hemorrhages in lungs, endocardium, brain, kidneys, urinary bladder, pancreas, and alimentary system. These results provide detailed insights into the gross pathology of wild boar infected with a highly virulent genotype II ASFV strain. From a didactic point of view, this detailed clinical course and macroscopic description may be essential for early postmortem detection of outbreaks in wild boar in the field and contribute to disease surveillance and prevention efforts.

## 1. Introduction

African swine fever (ASF) is a viral disease of domestic and wild suids currently threatening the global porcine industry [1,2]. Considered a notifiable disease to the World Organization for Animal Health (OIE), it has advanced remarkably in Africa, Europe, and Asia in recent years [3]. ASF is caused by an icosahedral, double-stranded DNA virus, the only member of the family Asfarviridae and the genus Asfivirus [4].

The current spread of African swine fever virus (ASFV) began in Georgia in 2007, from which it entered additional Caucasian countries, including the Russian Federation. In 2014, the virus progressed from the Russian Federation into the European Union via the Baltic countries (Lithuania, Latvia, Estonia, Poland). Between 2016 and 2019, eight more European countries notified the OIE of ASF infections: Moldova (2016), Romania (2017), Czech Republic (2017), Hungary (2018), Bulgaria (2018), Belgium (2018), Slovakia (2019), Serbia (2019) and Greece (2020) [3]. Since 2007, ASFV has affected domestic pigs and Eurasian wild boar (*Sus scrofa*); the latter are quite susceptible to disease, whereas other wild suids from Africa, such as warthogs (*Phacochoerus africanus*) and bush pigs (*Potamochoerus larvatus*), often do not develop clinical signs after infection [5].

In the European Union, ASFV currently affects primarily wild boar, which accounts for more than 90% of outbreaks in countries other than Romania, Bulgaria and Slovakia [3] The considerable increase in size and geographical distribution of wild boar populations makes them an increasingly important factor in the maintenance of the disease [6,7], which determines essential aspects of disease transmissibility, such as the contact rate between animals. Transmission of ASFV can be direct, i.e., between infected and susceptible animals, or indirect, such as between susceptible animals and virus present in infected carcasses or the environment, or carried by other potential vectors, such as ticks and flies [8,9,10].

Wild boar population has also been identified as an important factor in ASFV infection of domestic pigs. For example, ASF notifications on farms in Estonia correlate spatiotemporally with infections in wild boar, suggesting that wild boar is the main risk factor for infection of domestic pigs in this area [11]. In Sardinia, frequent contacts between wild boar and free-ranging pigs in certain areas of the island may help explain its persistent endemicity [12]. In fact, wild boar appears key to expanding ASFV infection, based on an anthropogenic and wild transmission cycle, which may involve ticks, for instance, in Africa and past outbreaks in the Iberian Peninsula [13].

Despite the central role of wild boar in current ASFV epidemiology, we are unaware of detailed studies of ASF pathology and course in this species with the ASFV strains currently circulating in Eurasia, in contrast to the details known about ASF infection in domestic pigs [5,6]. A detailed understanding of the disease in wild boar is necessary to ensure accurate differential diagnosis from other hemorrhagic diseases that can also affect these animals, including classical swine fever, porcine reproductive and respiratory syndrome, swine erysipelas, septicemic salmonellosis, as well as porcine dermatitis and nephropathy syndrome [4]. For this reason, a preliminary diagnostic of ASF based on the main gross lesions is an important key for early detection in new areas and may contribute widely to disease surveillance and control efforts [6].

To begin to understand ASF pathology and clinical course in wild boar, we experimentally infected animals with the Arm07 ASFV strain, which is highly virulent in domestic pigs and wild boar [14,15]. In addition, this strain is closely related to other strains isolated in Eastern Europe, such as Georgia 2007 strain [16,17]. Few studies have examined the pathology caused by closely strains in a minimal number of wild boars.

## 2. Results

### 2.1. Clinical Evaluation

A dose of 10 HAD_50_ of Arm07 strain was enough for the development of a clinical picture compatible with ASF disease that ended in a lethal outcome in all the six intramuscularly (IM) inoculated animals, but also in all the eleven in-contact animals, causing a similar clinical evolution and with the same lethal outcome.

**Figure 1:**
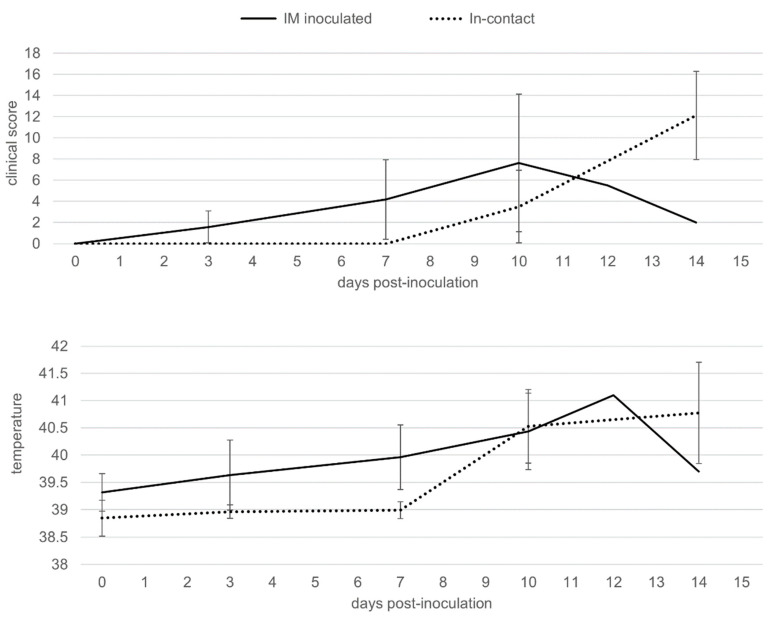
Average of clinical scores and body temperatures of wild boar intramuscularly (IM) inoculated with African swine fever virus (ASFV) Arm07 isolate (*n* = 6; solid line) and in-contact wild boar (*n* = 11; dashed line).

The first symptoms observed were nonspecific, such as fever, recumbence and loss of body condition. The six IM-inoculated animals started to show these first clinical signs from 3 to 6 dpi (4 ± 1 dpi) (Figure 1, Supplementary Tables S1 and S2), characterized by an increase in body temperature (40.5 ± 0.5 °C; 4/6 animals; 3–10 dpi) and reduced liveliness (5/6; 4–10 dpi). These symptoms were followed by localized or generalized erythema (6/6; 4–7 dpi), and slight walking difficulties (6/6; 4–5 dpi).

Meanwhile, the 11 in-contact animals started to show the first clinical signs from 9 to 11 dpi (10 ± 1 dpi), slightly later compared to the IM-inoculated animals (Mann-Whitney U test, *p* < 0.01), as expected, due to the incubation period. The clinical presentation in the 11 in-contact animals also began with nonspecific symptoms, characterized by an increase in body temperature (40.8 ± 0.7 °C; 10–14 dpi) and reduced liveliness in all in-contact animals (10–12 dpi) (Figure 1, Supplementary Tables S1 and S3). Other clinical signs were observed, including generalized erythema (5/11; 10–12 dpi), walking difficulties (7/11; 12–13 dpi), slight ocular discharge (9/11; 10–12 dpi), and digestive symptoms as feces with a large amount of mucus and sporadic vomiting (4/11; 10–14 dpi).

**Figure 2:**
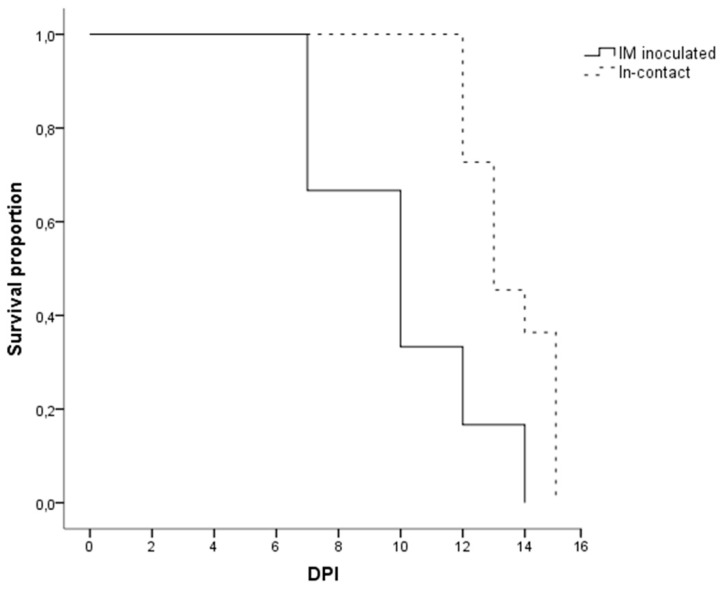
Kaplan-Meier curve showing the survival time in days post-inoculation (DPI) of the wild boar intramuscularly (IM) inoculated with ASFV Arm07 strain (*n* = 6; continues line) and the in-contact wild boar (*n* = 11; dashed line).

There were no statistically significant differences regarding the last clinical score (CS) recorded between the IM-inoculated (CS = 9 ± 6) and the in-contact animals (CS = 12 ± 4) (Mann-Whitney U test, *p* = 0.22). One out of the six IM-inoculated animals were euthanized, under the humane endpoint described below, and the rest were found dead, since they did not achieve the human endpoint criteria before succumbing to the disease, from 7 to 14 dpi (10 ± 3 dpi). Nine out of the eleven in-contact animals were euthanized, and the rest were found dead from 12 to 15 dpi (14 ± 1 dpi). In line with the delayed onset of symptoms, the in-contact animals succumbed to the disease slightly later than the IM inoculated animals (Mantel-Cox, χ2 = 7.44, 1 d.f.; *p* < 0.01) (Figure 2).

### 2.2. Postmortem Evaluation

**Figure 3:**
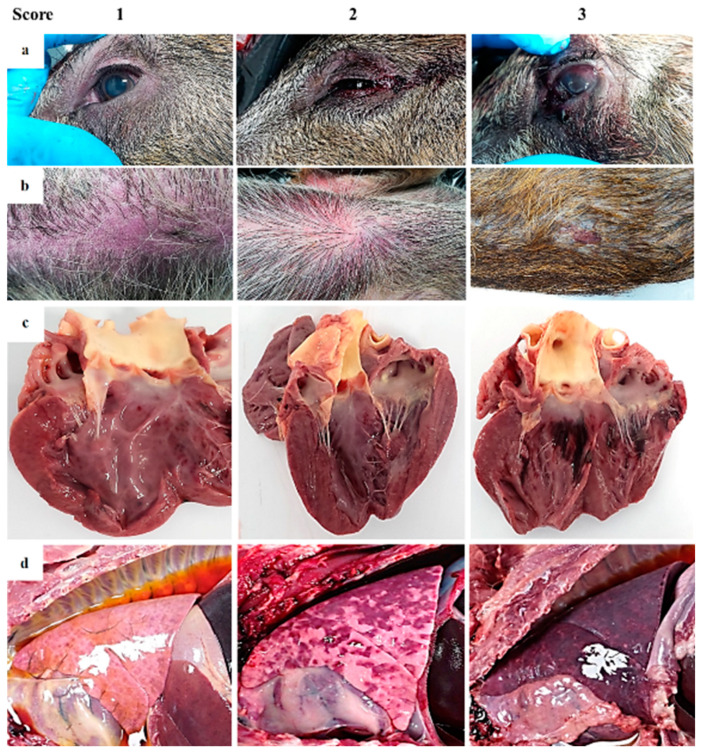
Guideline of macroscopic figures to evaluate the main pathological findings during the external examination and autopsy of wild boar infected with a highly virulent strain of African swine fever virus genotype II, Armenia 2007. (**a**) Conjunctiva and eyelids: Score 1—slight congestion; score 2—swelling of eyelids and serosanguineous ocular discharge; score 3—congested ocular mucosa with sclera hemorrhages, inflamed eyelids, and hyphemia. (**b**) Skin: Score 1—areas with skin erythema (ears, flanks, abdomen); score 2—erythema of the skin, multiple hemorrhages; score 3—erythema of the skin, multifocal to coalescent hemorrhages. Subcutaneous edema. Occasionally necrotic areas. (**c**) Heart: Score 1—petechial hemorrhages on endocardium; score 2—multifocal hemorrhages on the cardiac muscle and valvular endocardium; score 3—extensive coalescent hemorrhages on the cardiac muscle and valvular endocardium. A variable degree of valvular edema is observed in the atrioventricular valve. (**d**) Lung: Score 1—Absence of pulmonary parenchyma collapse associated with dilatation of interlobular septa, due to interstitial edema and the presence of some petechiae (interstitial pneumonia with severe pulmonary edema); score 2—patchy lobular consolidated areas characterized by slightly depressed areas with non-homogenous distribution (broncho-interstitial pneumonia); score 3—widespread lobe consolidation with a firm and dark red appearance, due to intense congestion and hemorrhages (lobar bronchopneumonia). Additional findings were thoracic effusion, septal edema and lack of pulmonary collapse.

In all animals from both groups of study, an autopsy revealed gross findings consistent with acute ASFV infection. External examination revealed a variable grade of congested ocular mucosa with hemorrhages, swelling (edematous) and congested eyelids (Figure 3a). Cutaneous lesions are characterized by marked erythematous areas, which were more visible in hairless zones, such as the abdominal wall and inguinal or axillary regions (Figure 3b). Some wild boar displayed multifocal to coalescent hemorrhages on the skin, and occasionally, necrotic areas.

Animals had moderate to severe accumulation of yellowish to reddish fluid in the abdominal cavity (ascites), thorax (hydrothorax) (Figure 3d) and pericardial sac (hydropericardium). Some animals displayed a bloody content with the presence of clots (hemoperitoneum, hemothorax and hemopericardium).

The mitral valve was slightly thickened, edematous and appeared whitish in all animals from both groups of study (edematous valve) (Figure 3c) and multifocal petechiae and ecchymosis were observed in the epicardium and myocardium layers, and valvular and parietal endocardium.

Grossly, lung (mainly caudal lobes) were prominent because of the absence of pulmonary parenchyma collapse. Most of the wild boar (11/17) displayed a dilatation of interlobular septa, due to interstitial edema, and the presence of some petechiae and small foci of lobular congestion (score 1). Lung surfaces showed a “mottled” appearance with multiple petechiae and ecchymosis, many of which had coalesced to cause extensive hemorrhage. These animals (4/17) showed multiple lobular consolidated areas characterized by slightly depressed areas with non-homogenous distribution compatible with broncho-interstitial pneumonia (score 2) associated with pleural adhesions or fibrinous pleuritis. Occasionally, widespread lobe consolidation was observed in two animals with approximately 90% of the lungs affected with a firm and dark red appearance (score 3), due to intense congestion and hemorrhages (Figure 3d).

**Figure 4:**
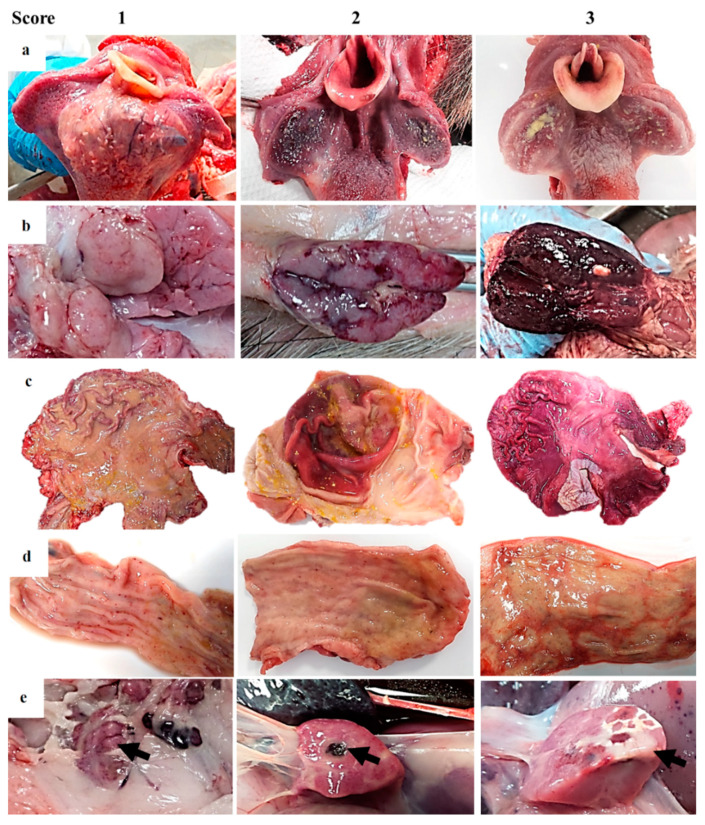
Guideline of macroscopic figures to evaluate the main pathological findings during the external examination and autopsy of wild boar infected with a highly virulent strain of African swine fever virus genotype II, Armenia 2007. (**a**) Tonsil: Score 1—erythematous; score 2—swelling and erythematous with fibrin on the surface; score 3—swelling and erythematous with fibrin/pus and multifocal necrosis on the surface. (**b**) Lymph node: Score 1—moderate color changes with occasional petechiae; score 2—petechiae and ecchymosis; score 3—increased size, edematous and hemorrhagic. Generalized lymphadenomegaly. (**c**) Stomach: Score 1—acute catarrhal gastritis; score 2—acute catarrhal gastritis with numerous petechiae/ecchymosis on the surface; score 3—hemorrhagic gastritis (more than 50% affected) with occasionally erosive lesions. (**d**) Intestine: Score 1—acute catarrhal enteritis with some petechiae on serosa surface; score 2—acute catarrhal enteritis with numerous petechiae/ecchymosis on serosa surface; score 3—hemorrhagic enteritis. I Pancreas: Score 1—multifocal hemorrhages on the surface; score 2—focal necrosis; score 3—extensive necrosis.

Several lymphoid tissues, including tonsils, spleen and lymph nodes, displayed intense changes in most animals. Tonsillar mucosa showed diffuse reddish discoloration, due to mild to moderate congestion. The tonsillar mucosa in one animal showed bilateral ulcerative necrosis and deposition of yellowish purulent material (bilateral necrotizing and suppurative tonsillitis) (Figure 4a). The spleen was dark red, and abundant blood oozed from the cut surface (congestion). The spleen was also markedly enlarged, with a relative weight range of 0.11–0.20 bodyweight (splenomegaly—normal value 0.13 ± 0.1 bodyweight [18]). Lymphadenomegaly was observed in all animals: Most lymph nodes (mandibular, retropharyngeal, tracheal, mediastinal, gastro-hepatic, renal, mesenteric, inguinal, splenic) were enlarged and intensely dark red and shiny, compatible with hemorrhagic lymphadenopathy (Figure 4b).

Moderate thickening of the digestive tract mucosa was diffusely observed. These thickened areas showed prominent edematous folds, abundant yellowish mucus, and multiple foci of small petechiae along the mucosal layer. The gastric mucosa showed multifocal erosions (superficial lesions), with normal tissue alternating with small, deep subacute ulcers (Figure 4c). The ileum, colon and especially rectum showed numerous hemorrhages (Figure 4d) associated with gut-associated lymphoid tissue (GALT) hyperplasia. Nine of the 17 animals showed necrotic areas at the edges of the pancreas (Figure 4e, arrow) and 7/17 multifocal petechiae on the surface.

**Figure 5:**
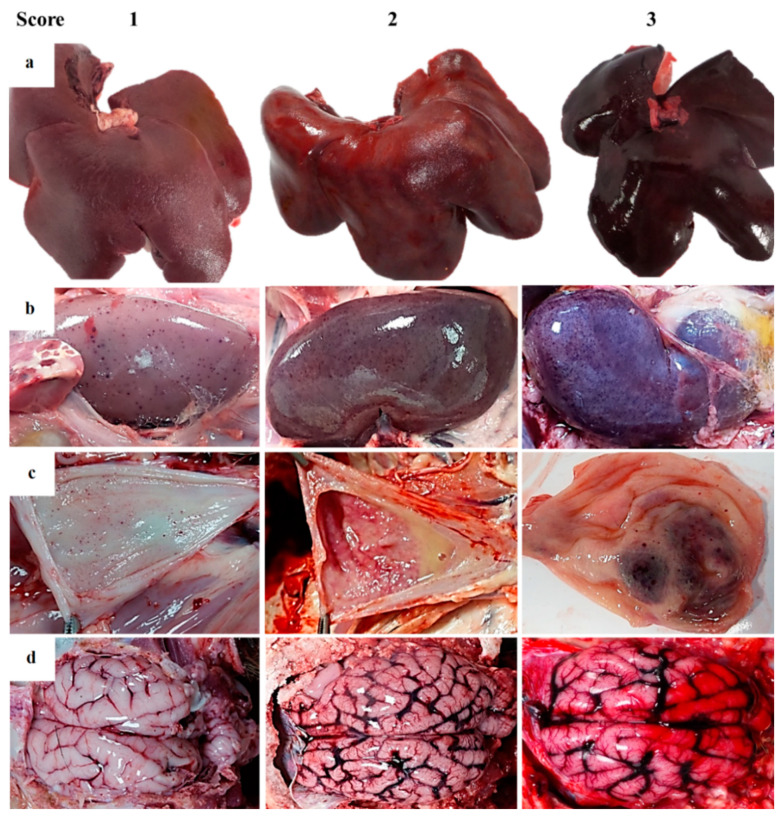
Guideline of macroscopic figures to evaluate the main pathological findings during the external examination and autopsy of wild boar infected with a highly virulent strain of African swine fever virus genotype II, Armenia 2007. (**a**) Liver: Score 1—mild multifocal parenchymatous color changes with slight hepatomegaly; score 2—moderate hepatomegaly, moderate congestion and hemorrhages. Mild diffuse lobular pattern; score 3—intense hepatomegaly, intense congestion and parenchymatous coalescent red zones; moderate panlobular pattern. (**b**) Kidney: Score 1—minimal to mild multifocal cortical and medullar petechiae; score 2—moderate multifocal cortical and medullar petechiae with a preponderance of cortical affectation; score 3—marked multifocal corticomedullar hemorrhages more frequent on the cortical layer with/without multifocal of moderate dark red pelvic areas. Perirenal edema. (**c**) Urinary bladder: Score 1—mild multifocal petechiae on mucosa surface with/without mild color changes in the urinary bladder wall; score 2—multifocal petechiae and ecchymosis on urinary bladder wall with edema. Turbid urine; score 3—multifocal to coalescent hemorrhages on the mucosal surface. Intense intramural edema. (**d**) Brain: Score 1—congestion of the meninges and/or edema; score 2—vascular congestion with occasional petechiae; score 3—severe vascular changes characterized by intense vascular congestion with focal hemorrhages of meninges (petechiae and ecchymosis).

The liver was enlarged in all animals (hepatomegaly) (Figure 5a). The hepatic parenchyma was reddish (congestion) with scattered pale foci, suggesting focal hepatic necrosis.

Kidneys showed moderate congestion upon incision. Multifocal spotted petechiae of up to 1–2 mm were observed on the cortex, as well as cortical and medullary parenchyma (Figure 5b). The renal pelvis showed severe edema, which in some animals was associated with hemorrhage and edematous perinephric fat (10/17). The mucosa of the urinary bladder showed different patterns of hemorrhages distribution. The majority of animals (11/17) displayed petechiae on the vesical mucosa in variable grade (Figure 5c). Six animals also displayed a thickening with irregular and prominent folds on the vesical mucosa, due to intramural hemorrhages; in these intense cases, urine contained numerous blood clots.

Submeningeal blood vessels of the brain were markedly engorged (severe vascular congestion) and causing numerous superficial and parenchymatous hemorrhages (Figure 5d).

The more frequent macroscopic lesions were generalized lymphadenomegaly (94.1%) and splenomegaly (76.5%); while the less frequent macroscopic lesions observed were: Acute catarrhal gastritis, edema in gallbladder wall, mild multifocal petechiae on mucosa surface in the urinary bladder, cardiac lesions (hydropericardium, valvular edema and numerous hemorrhages on heart layers), variable grades of fibrinous pleuritis, vascular congestion with focal hemorrhages of the meninges and multifocal necro-hemorrhagic lesions on the surface of the pancreas (see Table 1).

### 2.3. Tissue Sample Analysis

In all of the animals from the two different groups (IM inoculated and in-contact animals), ASFV DNA was detected in all 18 tissues evaluated. The cycle threshold (CT) values did not differ significantly between IM-inoculated or in-contact infected animals (Mann-Whitney U test, *p* = 0.331; Table 1). Mean CT values were significantly lower (indicating more abundant viral DNA) in the spleen (20.66 ± 4.90), liver (21.23 ± 4.19) and tonsils (21.73 ± 5.56) than in urinary bladder (25.94 ± 4.50) or intestine (27.26 ± 4.38) (Kruskal-Wallis, *p* < 0.01).

## 3. Discussion

In the present study, we have carried out an experimental infection with a low dose of a highly virulent ASFV isolate, Arm07, in wild boar. We have studied two different inoculation routes, IM inoculation and by contact route. The latest was performed in order to simulate a natural infection. In this way, we could evaluate the clinical progression of the disease and detail the main macroscopic pathological findings of these wild boar. Both groups developed similar clinical signs and succumbed to the disease, showing similar gross findings. The in-contact animals showed a delay in the onset and progression of the clinical disease regarding IM inoculated animals, as expected because of the incubation period.

The six IM inoculated wild boar succumbed from 7 to 14 dpi in contrast with the 5 dpi of survival time of the only previously reported case of a wild boar IM inoculated with another ASFV isolate of the current spread (Chechen Republic/09) [19]. This animal showed hemorrhagic lymphadenopathy, gastritis, pulmonary congestion and edema as gross findings during postmortem evaluation [18], which are in line with the lesions observed in the present infection study.

The infection of naïve wild boar by direct contact with infected animals has been reported before [19], in this study we have confirmed that wild boar IM infected with a low dose of 10 HAD_50_ is able to induce the similar clinical picture in-contact animals. The previous infection study with Chechen Republic/09 isolate also left three wild boar in-contact with the IM inoculated animal, that succumbed at 10 dpi, showing limited gross findings (hemorrhagic lymphadenopathy and gastritis) compared with the IM inoculated animal [19]. Meanwhile, our results show a survival time of the in-contact animals from 12 to 15 dpi, and the clinical course and the pathological findings, as well as the viral genome presence in tissues, did not differ between the different groups of study, suggesting that the infection route is not decisive for the clinical and pathological course.

These differences between studies could be explained by the different ASFV isolate used, the IM inoculation dose selected or the different ages of animals. However, it is important to take into account that our study has a high number of animals compared to previous infection studies in wild boar [19,20,21,22,23].

We observed lymphadenomegaly and splenomegaly in all wild boar, consistent with its high frequency in domestic pigs experimentally or naturally infected with other genotype II ASFV isolates from Caucasian region or Poland [22,24,25]. In general, lymphadenomegaly, hemorrhages in the lymph nodes, splenomegaly, and petechiae in several organs have been described as the main gross findings in domestic pigs [4,5].

Enlarged, hemorrhagic lymph nodes appear to be a characteristic of ASF, in both domestic pigs and wild boar [23,26]. This condition may be useful for characterizing ASF in free-ranging wild boar, in conjunction with less frequent macroscopic pathological findings but more specific, such as extensive hemorrhaging in vesical mucosa and necrotic foci in the pancreas, the latest findings have not characterized before in domestic pigs [6,26].

According to other authors, this state (along with ASFV infection) could induce an intense compromise the function of the immune system favoring the development of secondary infections and associated lesions [20,21]. For that reason, some of the gross postmortem lesions described in this study are not characteristic of acute clinical forms of ASFV infection, such as valvular edema, broncho-interstitial pneumonia, fibrinous pleuritic or pleural adhesions and necrotic tonsillitis. In this sense, further studies are needed to clarify these pathological findings related to secondary complications.

Domestic pigs seem to show greater vascular changes, such as edema and hemorrhages on the skin [4,5,26]. This type of cutaneous vascular disturbances is less striking on the wild boars. Meanwhile, erythematous areas in infected wild boar are occasionally observed, and only in areas devoid of hair or thin skin, such as abdominal and inguinal areas, it could be explained by the broader coat compared to domestic pigs.

Following clinical course description in pigs [5], we observed that the highest mortality in wild boar, due to high-virulent ASFV genotype II occurred within the subacute phase (within 15 days post-infection). Nevertheless, the gross lesions correspond to the acute course of infection in domestic pigs, according to Sánchez-Vizcaíno et al. [5]. Thus, many animals showed lesions more characteristic of the acute form of infection described in domestic pigs, such as cutaneous erythema, severe splenomegaly, hemorrhagic lymphadenopathy; and petechiae in the kidneys, urinary bladder, and heart (endocardium) [5]. Additionally, vascular changes (hemorrhages, edema, and congestion) characteristic of the subacute phase in domestic pigs, were appreciated: Severe enlargement of the spleen, due to congestion; effusion in corporal cavities (mild to moderate hydropericardium and ascites, and moderate to severe hydrothorax); and multiple hemorrhages (including petechiae and ecchymosis) in the lung, heart (endocardium), brain, kidneys, and urinary bladder [5].

Our studies provide more detailed information on ASFV infection in wild boar with virulent isolates from the current spread. As a recent review concludes, there are extensive studies on the genotype I ASFV isolates from the previous spread of this virus in the 1950s–1960s [20]. However, there is still a lack in terms of pathology of genotype II ASFV isolates from the current epidemical scenarios, and much less in wild boar [20], despite their important role in the epidemiology of the disease.

## 4. Materials and Methods

### 4.1. Virus

The highly virulent, hemadsorbing genotype II ASFV strain Arm07 was used in this infection experiment. The virus was propagated in porcine blood monocytes as described [15]. Viral titer was defined as the amount of virus causing hemadsorption in 50% of infected cultures (HAD50 per mL).

### 4.2. Animals

A total of 17 female wild boar 3–4 months old and weighing 10–15 kg were obtained from a commercial wild boar farm in Extremadura, Spain. These animals had not been vaccinated against any infectious disease and were antibody negative by ELISA test for the Spanish health surveillance infectious diseases in wild boar (RD 1082/2009) and also other diseases with important prevalence in Spain: Aujeszky virus, *Mycobacterium bovis*, classical swine fever virus, African swine fever virus, swine vesicular disease virus, *Mycoplasma pneumoniae*, porcine reproductive and respiratory syndrome virus and porcine circovirus type 2.

The animals were kept in the biosafety level 3 facilities of the VISAVET Health Surveillance Centre at the University Complutense of Madrid. Upon arrival, all animals were ear-tagged for individual identification, and they were first acclimated for two weeks before the study began. Throughout the study, animals had ad libitum access to food and water.

Animal experiments were conducted in strict accordance with national, regional and European regulations and the in vivo experimental protocol was approved by the Ethics Committees of the University Complutense of Madrid and Community of Madrid (PROEX 004/18 and 124/18). The protocol included a detailed description of efforts to prevent and avoid unnecessary suffering of the animals, including humane endpoints and the euthanasic guidelines. Briefly, the euthanasia was performed by intravenous injection of an overdose of narcotic substances (T61^®^, Laboratorios Intervet S.A., Salamanca, Spain) following anesthesia by intramuscular injection of tiletamine-zolazepam (Zoletil^®^, Virbac, Carros, France) and medetomidine (Medetor^®^, Virbac) as previously described [27]. All procedures were designed and performed by specifically trained and veterinarians (B, C, and D animal experimentation categories) according to EC Directive 86/609/EEC for animal handling and experiments.

### 4.3. Study Design

The study was performed to compare two different inoculation routes. In this sense, the 17 wild boars were divided into two groups. Six wild boars were intramuscularly (IM) inoculated with 1 mL of inoculum at a dose of 10 HAD50 of ASFV Arm07 isolate within the right semimembranosus muscle (IM group). The remaining 11 wild boar were left housed in direct contact with the IM inoculated animals throughout the study (in-contact group).

### 4.4. Clinical Evaluation

In order to describe, as accurately as possible, the evolution of the disease and be alerted to detect the reach of the humane endpoint by the animals, a clinical evaluation was daily performed. In this way, a 24-h video camera was used to monitor the wild boar and ensure their animal welfare and in situ wildlife-specialist veterinarian visits were conducted daily to record clinical signs.

The evolution of the ASFV infection was evaluated in terms of a quantitative clinical score (CS) following the specific clinical parameters for ASF disease in wild boar [27,28]. This CS includes rectal temperature, behavior, body condition, skin alterations, ocular/nasal discharge, joint welling, respiratory symptoms, digestive symptoms, and neurological symptoms. Fever was defined as a rectal temperature above 40.0 °C. Rectal temperature was the only clinical parameter that was not taken daily to minimize the management of animals, so it was only measured twice a week and in animals with any severe symptoms.

The humane endpoint was pre-defined as animals with a CS > 18, animals with severe clinical signs (level 4) of fever, behavior, body condition, respiratory and digestive symptoms for more than two consecutive days were also included, following the guidelines described by Cadenas-Fernández et al. [29]. In addition, animals that were suffering unacceptable conditions, based on veterinarian criteria, without reach the pre-defined humane endpoint were also euthanized.

### 4.5. Postmortem Evaluation

Gross findings were reported following the standardized macroscopic lesion guidelines for ASFV infection in domestic pigs previously described by Galindo-Cardiel et al., (2013) [30], with slight modifications, including more tissues evaluated and adapted to wild boar (see Table 2). During the external and internal survey, pathological alterations and its distribution from different tissues and organs were considered: External examination (body condition, eyes/conjunctiva and skin), thoracic, abdominal and pericardial contents, heart, lung, stomach, small and large intestine, liver and gallbladder, kidney, urinary bladder, spleen, lymph nodes (mandibular, retropharyngeal, tracheal, mediastinal, gastro-hepatic, renal, mesenteric, inguinal, splenic), tonsil, bone marrow, adrenal gland and brain.

### 4.6. Tissue Sample Collection and Analysis

During the postmortem examination, we have collected samples of blood, and from a wide selection of 18 sensitive tissues, eight of them were lymph nodes (renal, mediastinal, retropharyngeal, mesenteric, preescapular, gastrohepatic, inguinal and mandibular lymph nodes), as well as heart, liver, brain, spleen, palatine tonsils, lung, urinary bladder, kidney, bone marrow, and intestine. These tissue samples were analyzed in terms of viral genome detection by real-time PCR (qPCR) according to the protocol described by King et al. [32]. Viral DNA was extracted using the High Pure PCR Template Preparation Kit (Hoffmann-La Roche, Basel, Switzerland) as described by the manufacturer.

### 4.7. Statistical Analysis

The Mann-Whitney U test was performed to compare the average CS and the onset of clinical presentation in terms of days post-inoculation (dpi) between IM inoculated and in-contact infected animals. Kaplan-Meier survival curve and Mantel-Cox log-rank test were used to test significant survival differences between IM inoculated and in-contact infected animals. The cycle thresholds (CT) for ASFV DNA quantitation were analyzed to determine whether they differed significantly between IM-inoculated animals or in-contact infected animals (Mann–Whitney U test), or among the tissues sampled during autopsy (Kruskal–Wallis test). Calculations were performed in SPSS Statistics 20 (IBM, Chicago, IL, USA), and significance was defined as *p* < 0.05.

## 5. Conclusions

In general, the present detailed insights into lesions caused by the highly virulent ASFV isolate (Arm07) in wild boar may contribute to the early detection of the infection in this host in the field. In addition, this study confirms that the spleen and liver are the target tissues as samples of choice for passive and active surveillance in wild boar populations and laboratory diagnosis based on viral genome detection. Also, bone marrow could be a good tissue as a sample, since it has also shown low cycle thresholds results of PCR, and it has extra value because of its great interest in the field because this tissue could be a place where the viral genome was best preserved. However, further studies on it should be assessed. Finally, this study provides extensive and detailed guidelines specific for gross findings evaluation of ASF infection in wild boar, including several illustrations to standardize a scoring.

## Figures and Tables

**Table 1:** Frequency of main pathological findings and the mean and SD of the ASFV cycle threshold (CT) values for different tissues in six intramuscularly inoculated and 11 contact-infected wild boars. The gross scoring of pathological findings is described below in Table 2 in the materials and methods section.

	Tissue/Pathological Finding	*n* Animals Affected				ASFV CT Values ^1^	
		0	Score 1	Score 2	Score 3	IM	In-Contact
External examination							
	Body condition	0/17	14/17	3/17	0/17		
	Eyes/conjunctiva	0/17	8/17	4/17	5/17		
	Skin	0/17	4/17	12/17	1/17		
Thoracic cavity							
Cardiorespiratory system	Thoracic effusion	0/17	3/17	6/17	8/17		
	Heart					25 ± 7	23 ± 3
	a) Hydropericardium	0/17	9/17	6/17	2/17		
	b) Cardiac muscles	2/17	9/17	3/17	3/17		
	c) Edematous valve	0/17	9/17	6/17	2/17		
	Lung					22 ± 3	22 ± 2
	a) Collapse	0/17	0/17	5/17	12/17		
	b) Congestion/hemorrhage	0/17	11/17	3/17	3/17		
	c) Edema	0/17	1/17	8/17	8/17		
	d) Pneumonia	0/17	7/17	6/17	4/17		
	e) Pleura	4/17	9/17	2/17	2/17		
Abdominal cavity							
	Ascites (yellowish/reddish fluid)	0/17	7/17	7/17	3/17		
Alimentary system	Stomach	0/17	10/17	5/17	2/17		
	Small intestine	0/17	3/17	12/17	2/17		
	Large intestine	0/17	6/17	7/17	4/17		
	Liver	0/17	2/17	11/17	4/17	23 ± 7	20 ± 2
	Gallbladder	0/17	7/17	10/17	0/17		
	Pancreas	1/17	7/17	6/17	3/17		
Urinary system	Kidney (hemorrhages)					24 ± 5	22 ± 2
	a) Medullo-cortical pattern	0/17	5/17	8/17	4/17		
	b) Cortical-medullar pattern	0/17	7/17	8/17	2/17		
	Urinary bladder	1/17	10/17	6/17	0/17	26 ± 6	26 ± 3
Lymphoid system	Spleen	0/17	0/17	4/17	13/17	22 ± 6	20 ± 4
	Lymph nodes					25 ± 5 ^2^	23 ± 2 ^2^
	a) Lymphadenomegaly	0/17	0/17	1/17	16/17		
	b) Congestion/hemorrhages	0/17	3/17	4/17	10/17		
	c) Affected lymph nodes	0/17	1/17	5/17	11/17		
	Tonsils	0/17	5/17	5/17	7/17	25 ± 8	22 ± 7
	Bone marrow	0/17	17/17	0/17	0/17	24 ± 8	22 ± 4
Endocrine system—Adrenal glands		4/17	8/17	3/17	2/17		
Central nervous system—Brain		0/17	6/17	9/17	2/17	25 ± 7	23 ± 2
Blood						23 ± 7	18 ± 2

^1^ Mean cycle threshold (± standard deviation) in quantitative PCR of the indicated tissue in animals with the indicated pathological finding; ^2^ These CT values were obtained from a pool of eight different lymph nodes: Renal, mediastinal, retropharyngeal, mesenteric, preescapular, gastrohepatic, inguinal and mandibular lymph nodes.

**Table 2:** Gross scoring of pathological findings during the postmortem examination of wild boar infected with a highly virulent strain of African swine fever virus genotype II, Armenia 2007.

Tissue/Pathological Finding		Score		
		1 (Slight)	2 (Moderate)	3 (Intense)
External examination				
	Body condition	Slightly thin	Thin	Very thin
	Eyes/conjunctiva	Slight congestion	Congestion, swelling of eyelids and serosanguinous ocular discharge	Congested ocular mucosa with hemorrhages, inflamed eyelids and hyphemia
	Skin	Areas with skin erythema (ears, flanks, abdomen)	Erythema of the skin, multiple hemorrhages	Erythema of the skin, multifocal to coalescent hemorrhages. Subcutaneous edema. Occasionally necrotic areas
Thoracic cavity				
Cardiorespiratory system	Thoracic effusion	Moderate hydrothorax (with serosanguinous fluid)	Severe hydrothorax	Hemothorax
	Heart			
	a) Hydropericardium	Slight	Moderate with fibrinous adhesions	Intense with fibrinous adhesions
	b) Cardiac muscles	Petechiae on epicardium	Multifocal hemorrhages on the cardiac muscle and valvular endocardium	Extensive coalescent hemorrhages on the cardiac muscle and valvular endocardium
	c) Edematous valve	Slight thickening of the endocardium of the atrioventricular valves	Moderate thickening of the endocardium of the atrioventricular valves	Severe thickening of the endocardium of the atrioventricular and semilunar valves
	Lung			
	a) Collapse	Mild lack of collapse with no rib impressions	Moderate lack of collapse with mild or scarce rib impressions	Marked presence of foamy material in the trachea and intense distension of interlobular walls
	b) Congestion or hemorrhage	Mild diffuse/patchy congestion of parenchyma. No hemorrhages	Variable degree of congestion. Multifocal to coalescent randomly distributed petechiae or ecchymosis	Variable degree of congestion. Multifocal to coalescent random hemorrhages in pulmonary parenchyma
	c) Edema	Scarce or no presence of foamy material in trachea/bronchus (alveolar edema) and minimal (interstitial edema)	Mild to moderate presence of foamy material in trachea/bronchus and moderate distension of interlobular walls	Marked presence of foamy material in trachea/bronchus and intense distension of interlobular walls
	d) Pneumonia	Minimal to mild cranio-ventral (uni/bilateral) consolidation (bronchopneumonia)	Moderate cranio-ventral (uni/bilateral) consolidation (bronchopneumonia)	Marked-extended cranio-ventral (uni/bilateral) consolidation (bronchopneumonia) (with red and grey hepatization of lobules)
	e) Pleura	Fibrinous adhesions	Focal fibrinous pleuritis/pleural adhesions	Diffuse fibrinous pleuritis with or without pleural edema
Abdominal cavity				
Alimentary system	Ascites (yellowish/reddish fluid)	Slight	Moderate	Intense
	Stomach	Acute catarrhal gastritis	Acute catarrhal gastritis with numerous petechiae/ecchymosis on the surface	Hemorrhagic gastritis (more than 50% affected) with occasional erosions
	Small intestine ^1^	Acute catarrhal enteritis with some petechiae on serosa surface	Acute catarrhal enteritis with numerous petechiae/ecchymosis on serosa surface	Hemorrhagic enteritis with occasionally multifocal erosive lesions
	Large intestine ^1^	Acute catarrhal colitis/typhlitis/proctitis with some petechiae on serosa surface	Acute catarrhal colitis/typhlitis/proctitis with numerous petechiae/ecchymosis on serosa surface. GALT hyperplasia.	Hemorrhagic colitis/typhlitis/proctitis with occasionally multifocal necrotizing lesions associated with GALT hyperplasia
Alimentary system (cont.)	Liver	Mild multifocal parenchymal color changes with slight hepatomegaly	Moderate hepatomegaly, moderate congestion and presence multifocal red areas and hemorrhages. Mild diffuse lobular pattern	Intense hepatomegaly, intense congestion and presence of intra-parenchymal coalescent red zones. Moderate pan-lobular pattern
	Gallbladder	Mild to moderate edema affecting gallbladder wall/cystic duct. Normal bile	Edema affecting gallbladder wall/cystic duct with hemorrhages on serosa/submucosa surface. Bile with reddish aspect	Severe edema affecting gallbladder wall/cystic duct with severe hemorrhages. Bile content display red appearance with blood clots
	Pancreas	Multifocal hemorrhages on the surface	Focal necrosis	Extensive necrosis
Urinary system	Kidney (hemorrhages)			
	a) Medullo-cortical pattern	Mild multifocal cortical and medullar petechiae with multifocal vascular angiectasia	Moderate multifocal cortical (petechiae) and medullar (dark red band) hemorrhages with moderate pelvic dilation	Marked diffuse cortical-medullar hemorrhages with diffuse general renal dark red, marked pelvic dilation and extensive sub-capsular hemorrhages
	b) Cortical-medullar pattern	Minimal to mild multifocal cortical and medullar petechiae	Moderate multifocal cortical and medullar hemorrhages (petechiae) with preponderance of cortical affectation. Perirenal edema	Marked multifocal cortical-medullar hemorrhages more frequent on the cortical layer with/without multifocal of moderate dark red pelvic areas. Perirenal edema
	Urinary bladder	Mild multifocal petechiae on mucosa surface without/with mild color changes in the urinary bladder wall	Multifocal petechiae and ecchymosis on urinary bladder wall with edema. Turbid urine	Multifocal to coalescent hemorrhages on the mucosal surface. Intense intramural edema. Blood-stained urine with clots.
Lymphoid system	Spleen	Mild splenomegaly (minimal to mild bleeding after sectioning)	Moderate splenomegaly (moderate bleeding after sectioning)	Intense splenomegaly (marked bleeding after sectioning)
	Lymph nodes			
	a) Lymphadenomegaly	Slight	Moderate	Severe
	b) Congestion or hemorrhages	Moderate color changes with occasional petechiae	Petechiae and ecchymosis	Increased size, edematous and hemorrhagic
	c) Affected lymph nodes	Between 1–3 lymph nodes	Between 4–6 lymph nodes	6 or more lymph nodes
	Tonsils	Swelling and erythematous	Swelling and erythematous with fibrin on the surface	Swelling and erythematous with fibrin and multifocal necrosis on the surface
	Bone marrow	Congestion	Congestion with petechiae and ecchymosis	Congestion with multifocal to coalescent hemorrhages
Endocrine system—Adrenal glands		Multifocal petechiae hemorrhages in cortex and medulla	Medullar congestion. Petechiae and ecchymosis in cortex	Multifocal to coalescent hemorrhages predominantly in cortex
Central nervous system—Brain		Congestion meninges/edema	Congestion with occasional petechiae	Severe vascular changes with petechiae and ecchymosis

^1^ Catarrhal enteritis classified according to Maxie [31].

## Supplementary Materials

The following are available online at https://www.mdpi.com/2076-0817/9/9/688/s1, Table S1: Clinical evaluation, Table S2: Individual clinical score through the days post-infection of intramuscularly infected animals, Table S3: Individual clinical score through the days post-infection of contact infected animals.
