# Supplementary material for: Clinical Course and Gross Pathological Findings in Wild Boar Infected with a Highly Virulent Strain of African Swine Fever Virus Genotype II
Source: Pathogens. 2020 Aug 22;9(9):688. doi: 10.3390/pathogens9090688 (PMC7559345; doi:10.3390/pathogens9090688)
Supplement: Supplementary file 1 [file pathogens-09-00688-s001.pdf]

Supplementary Material

Table S1. Summary of individual clinical parameters of the in-contact and intramuscularly infected animals.

ID Animal	*Route of exposure*	*Onset of clinical signs (DPI)*	*Clinical score > 8 (DPI)*	*Last clinical evaluation recorded (CS)*	*Survival time (DPI)*	*Last blood sample analysis (CT)*	*Clinical signs*
1	In-contact	10	12	15	14	20	Fever, reduced liveliness, generalized erythema, moderate dyspnea, thick ocular discharge
2		10	-	7	13	24	Fever, generalized erythema, thick ocular discharge, transparent mucus in faeces
3		10	13	15	15	17	Fever, get up only to eat and drink, generalized erythema, slight walking difficulties only when getting up, significantly dyspnea
4		11	12	15	13	17	Fever, generalized erythema, moderate dyspnea, thick ocular discharge, vomiting < 24h
5		10	12	11	13	17	Reduced liveliness, generalized erythema, thick ocular discharge for more than two sequential days
6		10	11	16	12	16	Fever, get up only to eat and drink, generalized erythema, walking difficulties, joint swelling < 24h
7		9	12	17	15	18	Fever, get up only to eat and drink, generalized erythema, slight walking difficulties only when getting up, thick ocular discharge, transparent mucus in faeces

8		10	13	18	15	19	Fever, get up only touched, generalized erythema, walking difficulties, joint swelling, moderate dyspnea, slight ocular discharge, vomiting < 24h
9		9	10	17	12	17	Fever, get up only touched, generalized erythema, walking difficulties,
10		10	-	5	12	16	Reduced liveliness, localized slight erythema, slight walking difficulties
11		10	13	8	15	19	Reduced liveliness, localized erythema, slight dyspnea, slight ocular discharge
12	IM inoculated	4	7	17	10	15	Fever, get up only touched, generalized erythema, walking difficulties, joint swelling, moderate dyspnea, thick ocular discharge, transparent mucus in feces
13		3	6	8	7	22	Fever, get up only touched, generalized erythema, slight ocular discharge
14		6	-	5	12	33	Fever, localized erythema, anorexia
15		6	-	4	14	32	Fever, reduced liveliness
16		3	-	6	7	22	Fever, localized erythema, reduced liveliness
17		3	-	7	10	17	Fever, localized erythema, reduced liveliness, slight dyspnea

*CT = Cycle thresholds
*CS = Clinical score
*DPI = Days post-inoculation

Table S2. Individual clinical score through the days post-infection of intramuscularly infected animals. Including the time of onset of the following clinical signs: fever (F), lethargy (L), anorexia (A), skin alterations (S), ocular/nasal discharges (O), joint swelling (J), respiratory symptoms (R), digestive symptoms (D), neurological symptoms (N). Once a clinical sign was observed, it was maintained until the end of the experiment.

	Days post-infection															
ID	**0**	**1**	**2**	**3**	**4**	**5**	**6**	**7**	**8**	**9**	**10**	**11**	**12**	**13**	**14**	**15**
12	0	0	0	0*	1	1	2	9*	10	17*	17*/					
					L		A	S,O,R		J,D						
13	0	0	0	2*	4*	3	8	8+								
				F,S	L,A											
14	0	0	0	0*	0	0	3*	1*	2	2	5*	6*	5+			
							F		L,A			S				
15	0	0	0	0*	0	0	1*	0*	0	0	2*	1	2	4*	4+	
							F					L	A	S		
16	0	0	0	1*	1	1	6*	6+								
				L			F,A,R,O									
17	0	0	0	1*	1	1	3*	4*	2	7*	7+					
				L			F,L	A		R,O,D						

+ Found death / Euthanized *Clinical score including rectal temperature

Table S3. Individual clinical score through the days post-infection of contact infected animals. Including the time of onset of the following clinical signs: fever (F), lethargy (L), anorexia (A), skin alterations (S), ocular/nasal discharges (O), joint swelling (J), respiratory symptoms (R), digestive symptoms (D), neurological symptoms (N). Once a clinical sign was observed, it was maintained until the end of the experiment.

	Days post-infection															
ID	**0**	**1**	**2**	**3**	**4**	**5**	**6**	**7**	**8**	**9**	**10**	**11**	**12**	**13**	**14**	**15**
1	0	0	0	0*	0	0	0	0*	0	0	5*	3	11	15*	15*/	
											F,L,S		A,J,O,R			
2	0	0	0	0*	0	0	0	0*	0	0	3*	4	7	7+		
											F,L		S,O			
3	0	0	0	0*	0	0	0	0*	0	0	1*	3	7	10*	12*	15*/
											F		L,A,S,O			J,R
4	0	0	0	0*	0	0	0	0*	0	0	0*	7	10	15*/		
												L,A,S	O,R	D		
5	0	0	0	0*	0	0	0	0*	0	0	4*	7	11	11*/		
											F,L	A,S	O,R			
6	0	0	0	0*	0	0	0	0*	0	0	2*	8	16*/			
											F,L	A,S,J,O	D			
7	0	0	0	0*	0	0	0	0*	0	1	7*	6	8	15*	17*	17*/
											F,L,A,S		J,O	R,D		
8	0	0	0	0*	0	0	0	0*	0	0	1*	6	7	12	17*	18*/
											F	L,A,O	S	R,D	J	
9	0	0	0	0*	0	0	0	0*	0	2	12*	10	17*/			
											F,L,A,S, O,R,D		J			
10	0	0	0	0*	0	0	0	0*	0	0	5*	4	5+			
											F,L,J,O					
11	0	0	0	0*	0	0	0	0*	0	0	1*	2	4	8*	8*	8*/
											F		L,A,S	O,R		

+ Found death / Euthanized *Clinical score including rectal temperature
